# Comparative Biocompatibility of Portland‐Based Endodontic Cements Containing Niobium and/or Bismuth Oxides in Rat Subcutaneous Tissue

**DOI:** 10.1155/bmri/6614534

**Published:** 2026-04-01

**Authors:** Raphael Victor Silva Andrade, Rafaela Alcindo Silva de Souza Fé, Vladimir Galdino Sabino, Carlos Augusto Galvão Barboza, Maria Luiza Diniz de Sousa Lopes, Nizyara Costa da Silva, Valkleidson Santos de Araujo, Amanda Silveira da Silva, Raimundo Fernandes de Araujo Junior, Naianne Kelly Clebis, Isadora Luisa Gomes da Silva, Pedro Catalão Moura, Sofia Pessanha, Aurigena Antunes de Araújo

**Affiliations:** ^1^ Department of Dentistry, Federal University of Rio Grande do Norte (UFRN), Natal, Rio Grande do Norte, Brazil, ufrn.br; ^2^ Department of Pharmaceutical Sciences, Federal University of Rio Grande do Norte (UFRN), Natal, Rio Grande do Norte, Brazil, ufrn.br; ^3^ Department of Morphology, Federal University of Rio Grande do Norte (UFRN), Natal, Rio Grande do Norte, Brazil, ufrn.br; ^4^ Laboratory of Instrumentation, Biomedical Engineering and Radiation Physics (LA-REAL), NOVA School of Science and Technology, Caparica, Portugal; ^5^ Department of Biophysics and Pharmacology, Federal University of Rio Grande do Norte (UFRN), Natal, Rio Grande do Norte, Brazil, ufrn.br

**Keywords:** animal models, biocompatible materials, cell viability, endodontic materials, inflammation, subcutaneous tissue

## Abstract

**Objectives:**

To evaluate the in vitro cytocompatibility and osteogenic potential and the in vivo biocompatibility of two experimental endodontic cements based on Portland cement combined with either Nb2O_5_ (F6) and/or bismuth oxide (F7) in a rat subcutaneous implantation model.

**Methods:**

For the in vitro study, MC3T3‐E1 preosteoblastic cells were exposed to MTA Angelus, F6, or F7, and cell viability was assessed at 24 and 48 h using the Alamar Blue assay. Osteogenic differentiation was evaluated by alkaline phosphatase (ALP) activity after 14 days. For the in vivo analysis, polyethylene tubes containing the materials were implanted into the dorsal subcutaneous tissue of rats and evaluated at 7, 14, and 30 days, with empty tubes used as controls. Histological and histochemical analyses included hematoxylin and eosin (H&E), Picrosirius Red, Von Kossa staining, and TNF‐*α* immunohistochemistry. Chemical characterization was performed using Raman spectroscopy and X‐ray fluorescence (XRF). In vitro data were analyzed using one‐way ANOVA followed by Tukey’s post hoc test, while semiquantitative histological data were analyzed using the Kruskal–Wallis test followed by Dunn’s test. A significance level of 5% (*p* < 0.05) was adopted.

**Results:**

In vitro, F6 showed cell viability comparable to MTA Angelus at both 24 h (36.22 ± 2.66*%* vs. 35.75 ± 1.18*%*) and 48 h (34.18 ± 3.09*%* vs. 35.20 ± 2.35*%*) (*p* > 0.05). In contrast, F7 significantly reduced cell viability at 24 h (25.61 ± 1.11*%*) compared with MTA Angelus (*p* = 0.002), although no difference was observed at 48 h (*p* > 0.05). ALP activity after 14 days did not differ significantly among MTA Angelus (1.90 ± 1.23 UI/L/g), F6 (1.71 ± 0.88 UI/L/g), and F7 (1.78 ± 1.34 UI/L/g) (*p* = 0.703). In vivo, F6 induced mild to moderate inflammatory responses at the early time period, with a significant reduction by Day 14 and minimal inflammation at Day 30, comparable to MTA Angelus. No multinucleated giant cells were observed, and TNF‐*α* expression was moderate. Von Kossa staining and XRF confirmed calcium deposition, while Raman spectroscopy detected niobium species in peri‐implant tissues, indicating bioactivity. Picrosirius Red staining demonstrated progressive collagen fiber organization. Conversely, F7 elicited a pronounced and persistent inflammatory response characterized by multinucleated giant cells, intense TNF‐*α* expression, and bismuth retention in surrounding tissues, as confirmed by XRF, with no niobium‐related signals detected by Raman analysis.

**Conclusion:**

The niobium‐containing cement (F6) demonstrated favorable in vitro cytocompatibility and in vivo biocompatibility.

## 1. Introduction

Biocompatibility is a fundamental requirement in the development of new endodontic materials, especially those designed for direct contact with periapical tissues [1]. Mineral trioxide aggregate (MTA) is a widely used endodontic cement, primarily because of its excellent biocompatibility and hydrophilic nature. First introduced by Torabinejad et al. [2] in 1995,MTA is composed of a fine powder of trioxide containing tricalcium oxide, silicon oxide, and bismuth oxide, which hardens in the presence of moisture. The presence of hydrophilic particles such as tricalcium silicate and tricalcium aluminate further enhances their handling properties [3, 4]. Clinically, MTA is indicated for a variety of procedures, including apexification, apicoectomies with retrograde filling, and direct pulp capping [3].

There is extensive literature supporting the biocompatibility of MTA, particularly in relation to dentin and periodontal tissues [5, 6]. Its ability to stimulate the formation of apatite nucleation sites has been demonstrated in cultures of human osteoblast‐like cells [7]. This mineralization potential is largely attributed to the alkaline pH of the cement, as well as the release of calcium (Ca^2+^) and hydroxyl (OH^−^) ions during the setting process [8].

Bismuth oxide (Bi2O3) is the most commonly used radiopacifier in MTA [9, 10]. However, the incorporation of Bi2O3 into MTA impairs some physicochemical properties of the cement, including reduced stability, increased porosity, and the potential to cause dental staining [6, 11].

Considering these limitations, niobium pentoxide (Nb2O_5_) has been proposed as an alternative radiopacifying agent. Previous studies have demonstrated that Nb2O_5_ not only provides adequate radiopacity but also contributes to the formation of hydroxyapatite upon contact with biological tissues [12, 13]. Additionally, it has shown potential in enhancing the physicochemical properties of dental cements and promoting greater cell viability, indicating its potential for endodontic applications [12]. Then, we hypothesized that replacing Bi_2_O_3_ with Nb2O_5_ as a radiopacifier in Portland‐based cement would enhance tissue biocompatibility while maintaining its bioactive potential. Based on this evidence, the working hypothesis was that replacing Bi2O3 with Nb2O_5_ as a radiopacifier in Portland‐based cement could enhance tissue biocompatibility while preserving its bioactive properties.

Previously, our group obtained and characterized two formulations, F6 (Portland cement, Nb2O_5_, and calcium sulfate dihydrate [CaSO_4_·2H2O]) and F7 (Portland cement, Bi_2_O_3_, Nb_2_O_5_, and CaSO_4_·2H2O), which were compared with MTA (Angelus) [14]. In vivo biocompatibility analysis of rat subcutaneous tissue at 60 days demonstrated favorable outcomes for the F6 formulation, characterized by minimal inflammatory infiltrate and the absence of multinucleated giant cells, features not observed in the F7 group, which presented a more pronounced inflammatory response.

Thus, the null hypothesis of this study was that there would be no significant differences in biocompatibility, inflammatory response, or bioactivity between the experimental formulations (F6 and F7) and MTA at 7, 14, and 30 days.

Therefore, the aim of this study was to evaluate the biocompatibility of the experimental formulations F6 and F7 at 7, 14, and 30 days to gain a deeper understanding of their interaction with biological tissues.

## 2. Materials and Methods

### 2.1. Materials and Formulation of Experimental Cements

Commercial MTA Angelus was used as a reference material. Two experimental formulations were prepared: F6, consisting of Portland cement (Cimentos Apodi, Ceará, Brazil), Nb2O_5_ (CBMM, Araxá, Minas Gerais, Brazil), and CaSO4·2H2O (NEON, São Paulo, Brazil); and F7, containing Portland cement, Bi2O3 (NEON, São Paulo, Brazil), Nb2O_5_, and CaSO4·2H2O.

The Nb2O_5_ used was a highly crystalline white powder composed of ≥ 99.9% Nb2O_5_ with tantalum as the main impurity (< 1500 ppm). The material exhibited an orthorhombic/monoclinic phase, with a BET surface area between 3 and 5 m^2^/g. The particle size distribution included *D*_50_ ~ 40 *μ*m and *D*_90_ ~ 70 *μ*m, as well as a submicron fraction with *D*_50_ ranging from 0.6 to 2.0 *μ*m and *D*_90_ from 1.3 to 5.0 *μ*m. Thermal treatment above 900°C led to the transformation to the monoclinic phase.

CaSO4·2H2O was incorporated into the experimental formulations as a setting regulator and dimensional stabilizer. Its inclusion aimed to modulate the hydration kinetics of the Portland cement–based matrix, contributing to controlled setting behavior and improved handling characteristics. In addition, CaSO4 acts as a supplementary calcium source, which may favor early ionic exchange with the surrounding tissue without impairing the physicochemical stability or biocompatibility of the cement. The concentration of CaSO4·2H2O was selected based on previous formulations developed by our group, in which its presence did not negatively affect radiopacity, pH, or calcium ion release.

Details of the control and experimental cements and their corresponding starting reagents are presented in Table 1.

**Table 1:** Control and experimental cements.

MTA Angelus (Brazil)	Tricalcium silicate (3CaO·SiO2), dicalcium silicate (2CaO·SiO2), tricalcium aluminate (3CaO·Al2O3), calcium oxide (CaO), bismuth oxide (Bi2O3) as radiopacifier
F6	Portland cement (Cimentos Apodi, Ceará, Brazil) + calcium sulfate dihydrate (NEON, São Paulo, Brazil) + Nb2O_5_ (CBMM, Minas Gerais, Brazil)
F7	Portland cement + calcium sulfate dihydrate (NEON, São Paulo, Brazil) + Nb2O_5_ (CBMM, Minas Gerais, Brazil) + bismuth oxide (NEON, São Paulo, Brazil)

Using a ratio of 1:1 (0.14 g of powder:0.3 mL of distilled water), the formulations were manipulated with distilled water on a glass plate, with the aid of a #24 flexible metal spatula (SS White Durflex, Rio de Janeiro, Brazil), for 30 s, and the powder was gradually incorporated into the liquid until reaching the consistency of a gritty paste, following the same recommendations of the manufacturer of Angelus, which produces MTA. After manipulation, the samples were inserted into 3D printed molds previously produced to obtain specimens with standardized dimensions according to ISO 6876 and ANSI/ADA No. 57. The samples were subsequently stored at 37°C for 24 h.

For the in vitro assays, MC3T3‐E1 cells were cultured in 24‐well plates, each well presenting a growth area of 1.9 cm^2^. Cells were seeded at a density of 7 × 10^4^ cells per well, and one experimental sample was excluded from the analysis due to microbiological contamination, resulting in a final sample size of *n* = 4 or 5 per group.

Importantly, the group using only MTA (Angelus, Brazil) was also included in the in vitro analyses to serve as a reference material for comparison with the experimental formulations.

### 2.2. In Vitro Study

#### 2.2.1. Cell Viability Assay

MC3T3‐E1 preosteoblastic cells (American Type Culture Collection [ATCC], United States), derived from mouse calvaria, were cultured in 75‐cm^2^ flasks (TPP, Switzerland) with 7 mL of *α*‐MEM (Gibco, United States) supplemented with 10% fetal bovine serum (FBS) and 1% antibiotic–antimycotic solution (100 IU/mL penicillin, 100 *μ*g/mL streptomycin, and 0.25 *μ*g/mL amphotericin B). Cultures were maintained in a humidified incubator at 37°C with 5% CO2, with the medium replaced every 72 h. Subculturing was performed when the cells reached 75% confluence.

**Figure 1:**
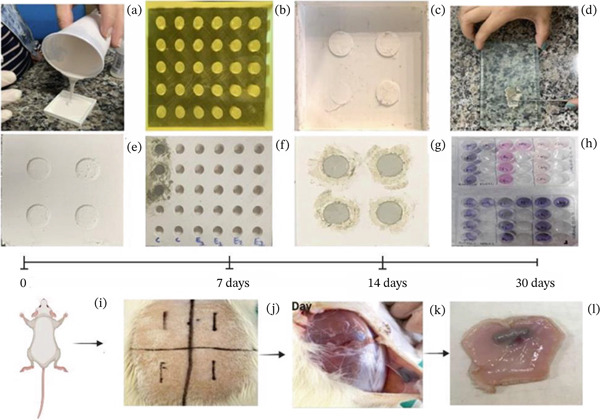
Cement preparation and experimental workflow for the study. (a–c) 3D molds used for fabricating the test samples. (d) Mixing and preparation of the cement formulations. (e–g) Fabrication of the test samples. (h) In vitro evaluation, including cell viability assays and alkaline phosphatase production. (i–l) Experimental model: biocompatibility of the implant materials. (i) Implant placement locations. (j–l) Collection of implants post euthanasia for analysis.

Cell viability was assessed at 24‐ and 48‐h intervals after irradiation, using the Alamar Blue assay (Invitrogen, United States), a colorimetric method that evaluates cellular metabolic activity through the reduction of resazurin (a blue, weakly fluorescent dye) to resorufin (a pink, highly fluorescent compound), which is mediated by the mitochondrial enzymes of viable cells. The cells were seeded in 24‐well plates at a density of 7 × 10^4^ cells/well. After cell seeding, the experimental specimens (MTA, F6, and F7) were placed into the culture medium of each corresponding group to evaluate their influence on cell metabolism. At each interval, the medium was replaced with 10% Alamar Blue diluted in *α*‐MEM, and the cells were incubated for 3 h. Supernatants were collected, and the absorbance was measured at 570 and 600 nm using a microplate reader (Epoch, BioTek Instruments, United States; Figure 1). Cell viability was calculated using the formula provided by the manufacturer.

#### 2.2.2. Alkaline Phosphatase (ALP) Activity

To assess osteogenic differentiation, ALP activity was measured after 14 days of cell culture on the material surfaces. The cells (7 × 10^4^) were seeded onto each disk/pellet and maintained under standard culture conditions. After 14 days, the culture medium was removed, the samples were washed with phosphate‐buffered saline (PBS), and the cells were lysed in 200 *μ*L of 0.1% Triton X‐100 (Merck, Germany) in assay buffer. ALP activity was quantified using a colorimetric assay (Labtest Diagnóstica, Brazil), which measures the conversion of p‐nitrophenyl phosphate to p‐nitrophenol. Reactions were set up with 20 *μ*L of lysate and 180 *μ*L of working reagent in 96‐well plates and incubated at 37°C for 90 min. Absorbance was measured at 405 nm using a microplate reader (Epoch, BioTek Instruments, United States). The total protein content of the lysates was determined using the Bradford method, with albumin used as the standard. ALP activity was interpolated from the absorbance values of a standard curve and normalized to the total protein content. A positive control group, which was cultured in osteogenic induction medium supplemented with 100 *μ*g/mL ascorbic acid and 5 mM *β*‐glycerophosphate (Merck, Darmstadt, Germany), was included [15].

### 2.3. In Vivo Study: Subcutaneous Biocompatibility

#### 2.3.1. Ethical Approval and Experimental Design

This preclinical in vivo study was approved by the Animal Ethics Committee for Teaching and Research (CEUA) of the Federal University of Rio Grande do Norte (UFRN) under protocol No. 037/2021, Certificate No. 267.037/2021, and was conducted between August and September 2024.

A total of 14 male Wistar rats (*Rattus norvegicus*), aged approximately 60 days and weighing between 280 and 380 g, were used. The animals were obtained from the Central Animal Facility of the Biosciences Center at UFRN. The rats were maintained under controlled environmental conditions, including a temperature of 22 ± 2°C, relative humidity of 45%–55%, and a standard 12/12‐h light/dark cycle. They had free access to filtered water and autoclaved rodent chow (Purina–Nuvilab CR‐1; Nuvital, São Paulo, Brazil).

The animals were randomly divided into three groups according to the observation periods: 7 days (*n* = 4), 14 days (*n* = 5), and 30 days (*n* = 5). The reduced number of animals in the 7‐day group is due to the death of one animal during the experimental period, prior to sample collection. Each animal received four subcutaneous implants on its dorsal region, including one implant from each experimental group, as described below, resulting in a total of 56 implants analyzed in the study.

The implants consisted of polyethylene tubes (Abbot Lab. of Brazil Ltda., São Paulo, São Paulo, Brazil), measuring 10.0 mm in length and 1.6 mm in internal diameter, in accordance with ISO 10993‐6:2007, and were sterilized with ethylene oxide (ISO 10993‐7:1995). Four implants were inserted into the dorsal subcutaneous tissue of each rat, with a minimum distance of approximately 20 mm between implants (center to center), distributed symmetrically on both sides of the dorsal midline to avoid overlap of local inflammatory responses.

The experimental groups were as follows: negative control (NC), represented by empty tubes; MTA, using MTA (Angelus, Brazil); F6, composed of Portland cement + Nb2O_5_ + CaSO4·2H2O; and F7, composed of Portland cement + Nb2O_5_ + Bi2O3 + CaSO4·2H2O.

The rats were anesthetized via intraperitoneal (IP) injection of ketamine (80 mg/kg) and xylazine (5 mg/kg). After shaving the dorsal area and performing antisepsis with 5% povidone‐iodine, four equidistant sites (4 cm apart) were marked on the back of each animal. At each site, an incision approximately 20 mm in length was made using a No. 15 scalpel blade (Fibra Cirúrgica, Joinville, Santa Catarina, Brazil), creating four subcutaneous pockets per animal (Figure 1).

The 56 polyethylene tubes, which were previously filled with the respective experimental materials, were subsequently implanted into the prepared subcutaneous pockets. The skin incisions were sutured using 3‐0 nylon thread (TECHNOFIO). Postoperative monitoring was conducted over a 24‐h period to prevent discomfort or complications. Analgesia was provided with ibuprofen at a dose of 15 mg/kg/day, which was administered for three consecutive days in the drinking water (2.5 mL of a 100 mg/5 mL solution diluted in 500 mL of water).

Euthanasia was performed at the predetermined time period of 7, 14, and 30 days post implantation using a lethal IP dose of ketamine (240 mg/kg) and xylazine (30 mg/kg). Following euthanasia, the dorsal area was shaved again, and a longitudinal incision was made using the vertebral column as a midline reference. Each implant was located and excised together with the adjacent tissues. Both open ends of each polyethylene tube were filled with the respective cement formulations. After explantation, the samples were carefully oriented, and each end of the tube was assigned to a specific analytical procedure. One end of the implant, together with the surrounding tissue, was fixed and processed for histopathological analyses, whereas the opposite end was preserved in 70% ethanol and sent to Lisbon (Portugal) for Raman spectrometry/two‐dimensional energy‐dispersive X‐ray fluorescence (2D‐EDXRF) analyses.

The samples were fixed in 10% paraformaldehyde solution prepared in 0.1 M PBS for 48 h. After fixation, the samples were processed and embedded in paraffin blocks for histological analysis, including hematoxylin and eosin (H&E) staining, Von Kossa staining, polarized light microscopy, Picrosirius Red (PSR) staining, and immunohistochemical staining.

Raman spectrometry and 2D‐EDXRF were analyzed directly on the tissue–material interface to preserve the native chemical composition.

#### 2.3.2. H&E Staining

From the paraffin‐embedded samples, histological sections of 5‐*μ*m thickness were obtained in serial sagittal planes. The resulting sections were stained with routine H&E and examined under a conventional light microscope (Olympus CH2, Olympus Optical Co., Ltd., Japan) by a single, blinded pathologist who was unaware of the experimental groups. Inflammatory infiltration was assessed in the tissue area in direct contact with the exposed end of the implanted tubes at 400× magnification and classified at the three experimental time periods (7, 14, and 30 days) as follows [16]: (1) no or few inflammatory cells and no reaction; (2) fewer than 25 cells and mild reaction; (3) between 25 and 125 cells and moderate reaction; and (4) 125 or more cells and severe reaction. The presence of necrosis and multinucleated giant cells was also evaluated, as well as the thickness of fibrous capsules, which were classified as thin (< 150 *μ*m) or thick (≥ 150 *μ*m) in the tissue adjacent to the implanted material [16].

#### 2.3.3. Von Kossa Staining and Polarized Light Analysis

The paraffin‐embedded samples were sectioned at 5‐*μ*m thickness in serial sagittal planes and mounted on glass slides. For Von Kossa staining, the sections were treated with 5% silver nitrate solution and exposed to natural sunlight for 15–30 min. After rinsing, the slides were immersed in 5% sodium thiosulfate for 5 min and subsequently counterstained with safranin for 5 min.

The presence of calcium deposits was assessed under a conventional light microscope (Olympus CH2, Olympus Optical Co., Ltd., Japan) by a blinded pathologist. Sections showing black or dark brown areas were considered positive for calcification [17].

For polarized light analysis, unstained sections were examined using a Leica DM750 polarized light microscope (Leica Microsystems) equipped with a Leica EC3 digital camera. Images were captured at 200× magnification and processed with Leica Application Suite LAS EZ v1.6.0 software. Calcification was recorded as either present or absent [17], and the optical properties of the cement materials within the biological tissue were qualitatively evaluated using multipolarized light (MPL) imaging.

#### 2.3.4. PSR Staining

To quantify the area occupied by collagen fibers, histological sections were stained with 0.1% PSR and analyzed under an optical microscope (BX51, Olympus) equipped with a polarized light filter. Five animals per group were evaluated, and two images were captured per implant, resulting in a total analyzed area of 2.788 mm^2^ per group. Images were obtained at 200× magnification. Collagen quantification was performed via Image‐Pro Plus 6.0 software (Ipwin 32, Media Cybernetics, Maryland, United States). In each microscopic field, the collagen fiber area was segmented by hue (Measure > Count/Size > Manual Select Colors > Precision 1 × 1 > Histogram‐based > Color Frequency: red 230–256; green 52–128 > New Mask > Close > Automatic Bright Objects > Count > View > Statistics > Copy Sum). Collagen types are identified by color: red corresponds to Type I collagen, and green corresponds to Type III collagen [18, 19]. The results are expressed as the percentage area of each collagen type per image (field area: 1.394 mm^2^) [20].

#### 2.3.5. Immunohistochemistry

Paraffin‐embedded tissue sections (3‐*μ*m thick) from Day 7 samples were obtained, mounted on silanized slides, and incubated at 37°C for 48 h. After deparaffinization and rehydration, antigen retrieval was performed using citrate buffer (pH 6.0) in a water bath (90°C, 15 min). Endogenous peroxidase activity was blocked with 3% H2O2, and nonspecific binding was prevented with SuperBlock (Thermo Fisher). The sections were then incubated overnight at 4°C with an anti‐TNF‐*α* primary antibody (Boster Biological Technology, Pleasant, California, United States; catalog #PA1079; dilution, 1:500). The following day, the slides were washed with PBS and incubated with HRP‐conjugated goat anti‐rabbit IgG (Boster Biological Technology, Pleasant, California, United States; catalog #BA1054). After the samples were washed twice with PBS, immunoreactivity was visualized using DAB (EasyPath). Counterstaining was performed with Mayer’s hematoxylin, followed by dehydration and mounting. TNF‐*α* immunoreactivity was assessed semiquantitatively by a pathologist who was blinded to the experimental groups under light microscopy (100× magnification). Positive immunostaining was identified by brown cytoplasmic or extracellular matrix labeling. Both cellular and extracellular matrix immunoreactivity were considered relevant. The scoring system ranged from 1 to 4: (1) no immunoreactive cells; (2) low immunoreactivity (few positive cells and weak matrix labeling); (3) moderate immunoreactivity (moderate number of positive cells and matrix labeling); and (4) high immunoreactivity (numerous positive cells and strong matrix labeling) [21].

#### 2.3.6. Tissue Analysis by Raman Spectrometry

Subcutaneous tissue samples (*n* = 3 per group) were collected after 30 days of experimental implantation. These samples were preserved in 70% ethanol and subsequently sent to the Department of Physics at NOVA University of Lisbon for the analysis of Bi^3+^ or Nb^5+^, [NbO4]^3−^ (niobate), or [NbO6]^7−^ (hexaniobate). A confocal Raman XploRA (Horiba) system with a 785‐nm laser and CCD detector was used. Using a 100× objective and a 50% neutral density filter, we obtained a sample power of 5.0 ± 0.4 mW (LaserCheck, Edmund Optics). This system allows the examination of samples within a spectral range of 300 to 1800 cm^−1^ with a resolution of 4 cm^−1^.

#### 2.3.7. Tissue Analysis via 2D‐EDXRF

The same subcutaneous tissue samples described in Section 2.3.6 (*n* = 3 per group) were used for this analysis. A benchtop micro‐XRF (X‐ray fluorescence) spectrometer, the M4 TORNADO by Bruker (Berlin, Germany), was used. The system uses a low‐power X‐ray tube with an Rh anode operated at 50 kV and 300 *μ*A and a polycapillary lens that focuses the beam on a 40‐*μ*m spot for the Mo‐K*α* emission lines. To minimize eventual contamination due to the presence of atmospheric elements such as argon (Ar) and improve detection limits, the analysis chamber was kept at a vacuum pressure of 20 mbar. Additionally, a combination of 100‐*μ*m Al/50‐*μ*m Ti/25‐*μ*m Cu filters was used between the X‐ray tube and the samples to improve the signal‐to‐noise ratio of the lower region of the spectrum. The map analyses were performed using an area of interest defined in the software regarding each of the analyzed samples, and scanning measurements were performed on the selected areas with a step size of 40 *μ*m and a 10‐ms acquisition time per pixel. Additionally, point measurements were performed at the desired spot to determine the elemental composition of the tissue.

### 2.4. Statistical Analysis

The data were tabulated and exported to the statistical software GraphPad Prism 8.01 (GraphPad Software, San Diego, California, United States). For the in vitro experiments (cell viability and ALP activity), data that followed a normal distribution (Shapiro–Wilk test) were analyzed using one‐way ANOVA followed by Tukey’s post hoc test for intergroup comparisons, and paired *t*‐tests were applied for intragroup comparisons (24 vs. 48 h). After descriptive analysis was performed, for the semiquantitative score data (H&E, PSR, and immunohistochemistry), comparisons among groups were performed using the Kruskal–Wallis test followed by Dunn’s post hoc test. A significance level of 5% (*p* < 0.05) was adopted for all analyses.

## 3. Results

### 3.1. In Vitro Analysis

#### 3.1.1. Cell Viability

The percentages of viable cells in the studied groups are shown in Table 2.

**Table 2:** Mean ± standard deviation of the viability (%) of MC3T3‐E1 cells exposed to the tested formulations compared with MTA (*n* = 5).

Cell viability		
Group	24 h	48 h
MTA	35.745 ± 1.179	35.197 ± 2.349
F6	36.221 ± 2.655	34.180 ± 3.093
F7	25.611 ± 1.112^∗^	34.602 ± 1.112

^∗^p = 0.002 (F7 compared with MTA).

A statistically significant difference was observed for formulation F7 compared with MTA at the 24‐h time period (p = 0.002).

#### 3.1.2. ALP Activity

The mean and standard deviation (SD) values for the osteogenic capacity of formulations F6, F7, and MTA, which were determined via the ALP activity assay, are presented in Table 3. No statistically significant differences were detected among the MTA, F6, and F7 groups (*p* > 0.05).

**Table 3:** The mean ± standard deviation of alkaline phosphatase activity in preosteoblastic cells (MC3T3‐E1) after 14 days of osteogenic induction, expressed as (UI/L)/protein (g). Comparison with MTA (*n* = 5; *p* > 0.05).

Group	Alkaline phosphatase activity (UI/L)/protein (g)
MTA	1.900 ± 1.227
F6	1.713 ± 0.8831
F7	1.784 ± 1.342

### 3.2. In Vivo Analysis: Subcutaneous Biocompatibility

#### 3.2.1. Inflammation Analysis

**Figure 2:**
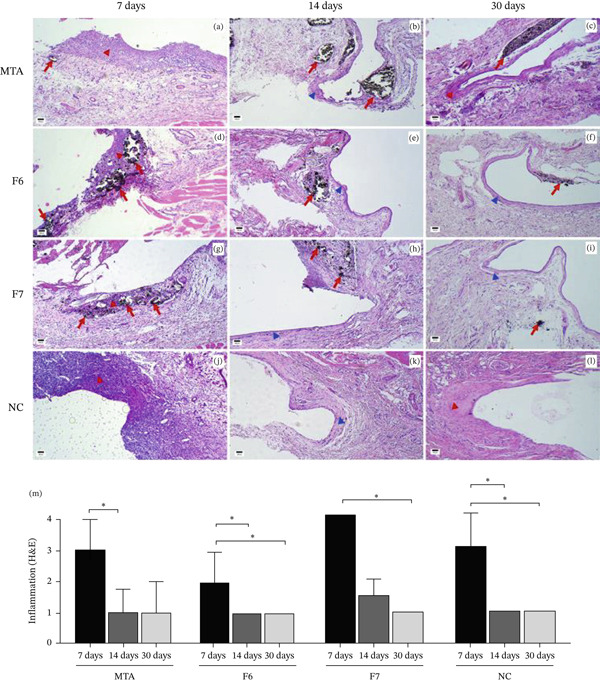
Representative photomicrographs of the subcutaneous reactions in H&E‐stained samples from the MTA, F6, F7, and negative control (NC) groups. (a, d, g, j) Moderate to intense inflammatory infiltrate and thick fibrous capsule (red arrowheads) in the specimens evaluated at 7 days. (b, e, h, k) Absent to mild inflammatory infiltrate and thin fibrous capsule (blue arrowheads) at 14 days. (c, f, i, l) Absent inflammatory infiltrate at 14 days. Dark particles (red arrows) were evident in the MTA, F6, and F7 groups at all time periods (H&E, 100×; scale bar: 100 *μ*m). (m) Bar graph illustrating the inflammation scores. Bars represent the median, and the error bar indicates the interquartile range. The Kruskal–Walli test revealed no difference between the groups at any time period ( ^∗^Dunn’s test; *p* < 0.05)

Figure 2 shows the results of the histological analysis of the MTA (Figure 2a,c), F6 (Figure 2d,f), F7 (Figure 2g,i), and NC (Figure 2j,l) groups at 7, 14, and 30 days.

Histological evaluation demonstrated that collagen deposition was consistently restricted to the fibrous capsule surrounding the tube, which corresponds to the typical foreign body response to the implanted carrier. Importantly, no collagen deposition or fibrotic reaction was observed in the surrounding tissue adjacent to extruded material at any experimental time period.

On Day 7, inflammation was observed in all the samples, with most evaluated as moderate inflammation in the MTA, F6, and CN groups and severe inflammation in the F7 group, without statistically significant differences among the groups (*p* = 0.287). A predominance of mononuclear inflammatory cells was observed, along with some polymorphonuclear cells. A thick fibrous capsule (≥ 150 *μ*m) was evident in all the samples analyzed across the different groups at 7 days (Figure 2a,d,g,j).

On Day 14, most specimens exhibited mild inflammation regardless of the group. A more pronounced presence of lymphocytes and plasma cells was noted, but there was no significant difference between the groups (*p* = 0.560). Thin capsules were observed in the evaluated tissues of all groups (< 150 *μ*m), with only one specimen from the F7 group presenting a thick capsule.

After 30 days, all the groups presented similar features, such as the absence of inflammation or the presence of only sparse inflammatory cells (*p* > 0.999). In all the groups, thin capsules were predominant (< 150 *μ*m). A thick capsule was observed in only one specimen from the NC group.

According to the intragroup analysis, a progressive reduction in the degree of inflammation was observed over time. The results of the Kruskal–Wallis test revealed that this reduction was statistically significant for the MTA (*p* = 0.026), F6 (*p* = 0.016), F7 (*p* = 0.007), and NC groups (*p* > 0.028; Figure 2).

Necrosis was observed in only one specimen from the NC group on Day 7. Giant cells were identified exclusively in the F7 group, with one specimen on Day 7 and two specimens on Day 14.

#### 3.2.2. Calcium Deposition Analysis

**Figure 3:**
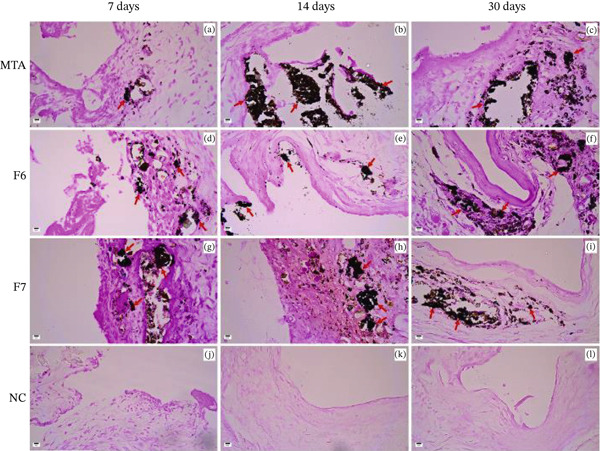
Representative photomicrographs illustrating Von Kossa staining of specimens from the (a–c) MTA, (d–f) F6, (g–i) F7, and (j–l) NC groups at 7, 14, and 30 days. Blackened structures indicative of dystrophic calcification (red arrows) were observed at all time periods in all groups except the NC group. 200× magnification.

Subcutaneous sections containing the implants with MTA, F6, and F7 exhibited positive Von Kossa staining, characterized by black‐stained precipitates indicative of dystrophic calcification, across all evaluated time periods (Figure 3a,i). Unlike H&E staining, which allows the assessment of general tissue morphology and inflammatory response, Von Kossa staining specifically identifies phosphate‐ or carbonate‐bound calcium salts, appearing as black deposits and confirming the presence of mineralized calcium precipitation rather than cellular or structural components.

At 7 days, small and sparsely distributed calcified deposits were observed in the connective tissue adjacent to the implants. At 14 and 30 days, the stained areas became more extensive and well defined, with larger and more intense dystrophic calcification deposits predominantly localized near the implant–tissue interface. Importantly, these calcified deposits were not associated with organized mineralized tissue formation but rather with localized calcium precipitation, consistent with bioactive material–tissue interaction.

The NC group, which received polyethylene tubes without any formulation, showed no evidence of Von Kossa–positive staining at any of the evaluated time periods (Figure 3j,l), confirming the absence of calcium deposition in the control condition.

**Figure 4:**
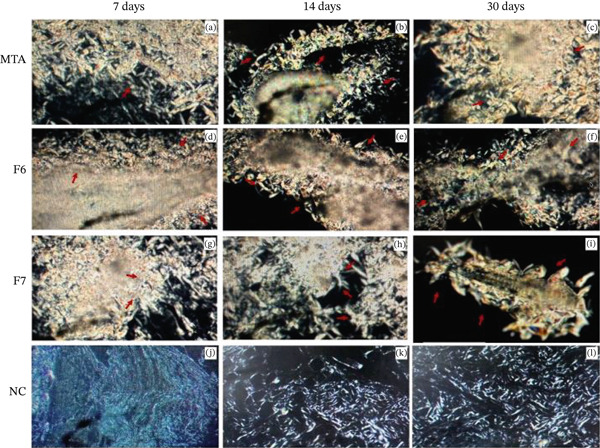
Representative images of the samples analyzed at 7, 14, and 30 days under polarized light. Groups showing birefringent depositions (red arrows): (a–c) MTA, (d–f) F6, and (g–i) F7. (j–l) Negative control group showing no birefringent structures under polarized light. Polarized light visualization: 200×.

Polarized light analysis demonstrated that tissues exposed to implants containing MTA, F6, and F7 cements presented areas with crystalline habits and birefringence characteristic of calcium crystals (Figure 4a,l). In contrast, the NC group implanted with polyethylene tubes alone, without any cement formulation, showed no birefringence or features suggestive of calcium crystals (Figure 4a,c).

#### 3.2.3. PSR Analysis

**Figure 5:**
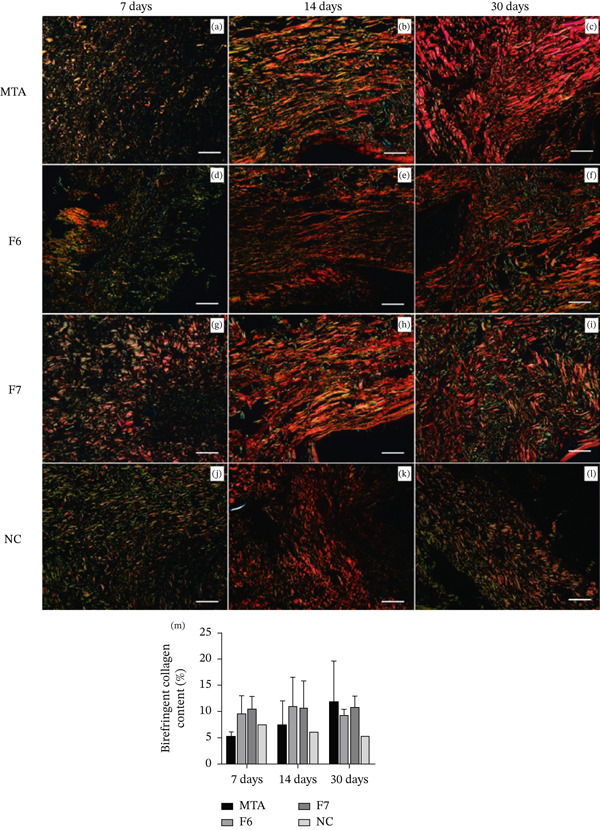
Representative images of subcutaneous tissue stained with Picrosirius Red under polarized light microscopy at (a–l) 7, 14, and 30 days and (m) quantitative analysis of birefringent collagen content (%). The images show the presence of collagen fibers with varying degrees of birefringence across all groups and time periods. Bars represent the means ± standard deviations for each experimental group (MTA, F6, F7, and NC) at each evaluation period. Two‐way ANOVA revealed no statistically significant differences among groups, time periods, or their interactions (*p* > 0.05). Scale bar: 50 *μ*m. Polarized light: 200×.

After 7 days, fine birefringent collagen fibers were observed in the capsule tissues of all the groups (Figure 5a,d,g,j), with a predominance of greenish staining and loosely organized fibers. At 14 days, all the groups presented increases in collagen fiber density and thickness, with more evident thick and organized collagen bundles displaying reddish birefringence (Figure 5b,e,h,k). By 30 days, the collagen pattern continued to intensify, with thicker and more organized fibers predominating across all groups (Figure 5c,f,i,l).

A time‐related progression in collagen deposition was visually noticeable, particularly in the F6 and F7 groups at 14 and 30 days and the NC group from 14 days onward. However, the quantitative analysis did not reveal statistically significant differences among groups, time periods, or their interaction (*p* > 0.05; Figure 5m).

#### 3.2.4. Immunoexpression of TNF‐*α*

TNF‐*α* showed a predominantly cytoplasmic immunostaining pattern. At 7 days, TNF‐*α* immunoexpression was evident in all the experimental groups (Figure 6). The F7 group presented the highest scores, indicating a more pronounced proinflammatory effect, whereas both the MTA and F6 groups presented moderate and comparable TNF‐*α* expression levels. The NC group presented lower TNF‐*α* immunostaining, with greater variability among the samples, as expected for the baseline inflammatory condition. A significant difference among the groups was observed (*p* = 0.0364), with pairwise comparisons showing that the F7 group had significantly higher TNF‐*α* expression than the NC group (*p* = 0.0406).

Figure 6Immunohistochemical expression of TNF‐*α* in the different experimental groups at 7 days. (a) MTA, (b) F6, (c) F7, and (d) NC. (e) The bars in the graph represent the medians and interquartile ranges. Statistical analysis using the Kruskal–Wallis test revealed significant differences among the groups (*p* = 0.0364).  ^∗^Dunn’s post hoc test showed that the F7 group presented significantly higher TNF‐*α* expression than the negative control group (*p* = 0.0406). Scale bar: 100 *μ*m.

**Figure:**
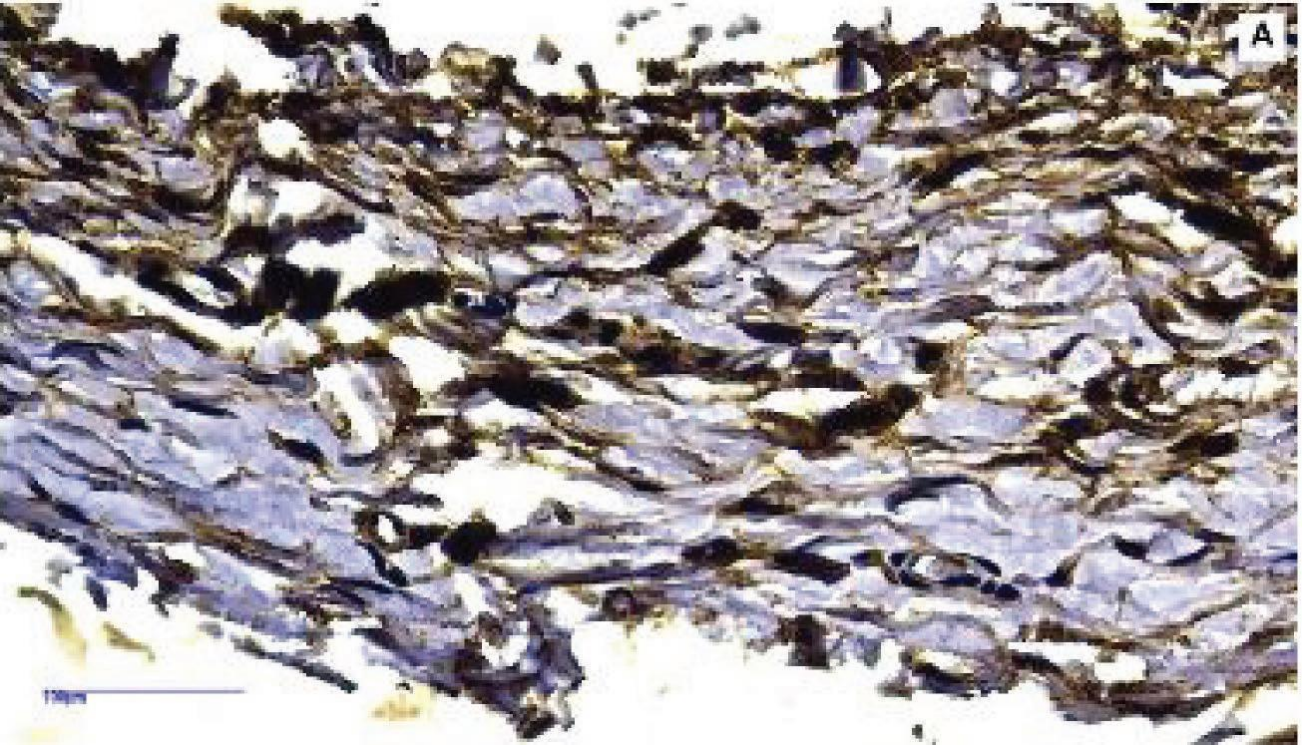
(a)

**Figure:**
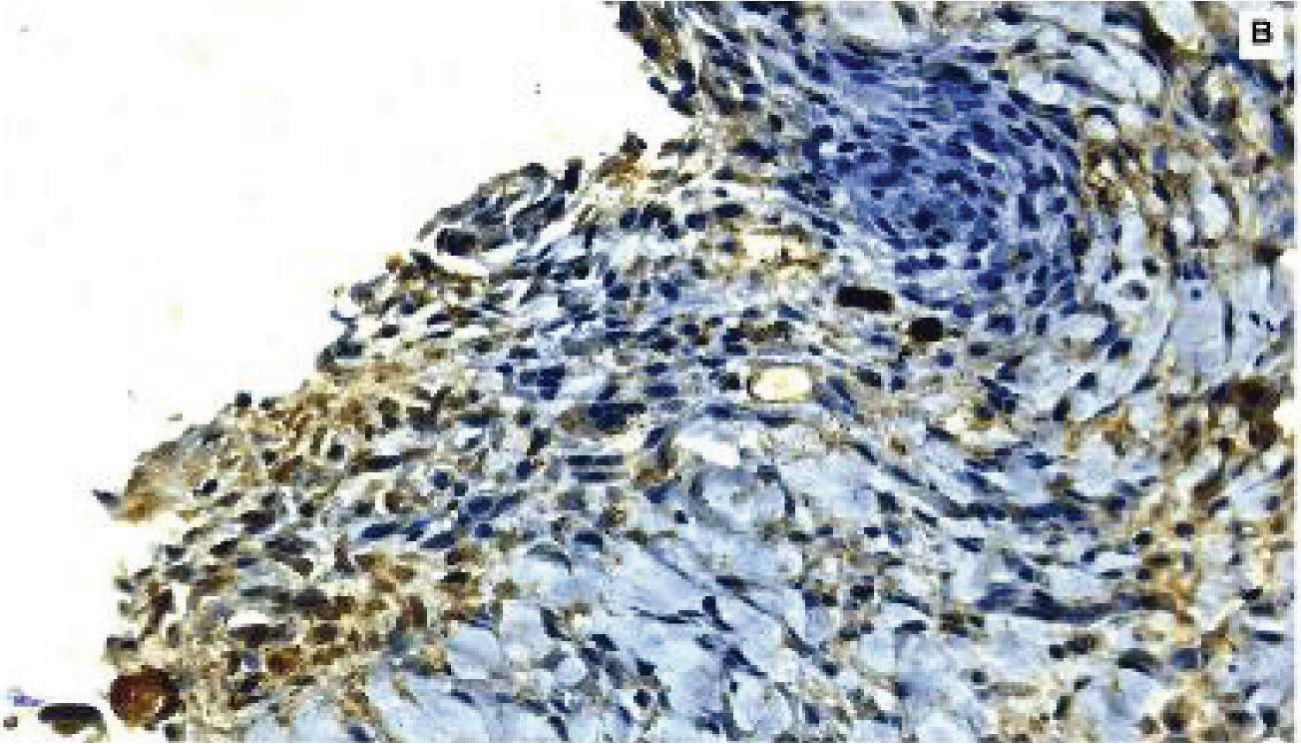
(b)

**Figure:**
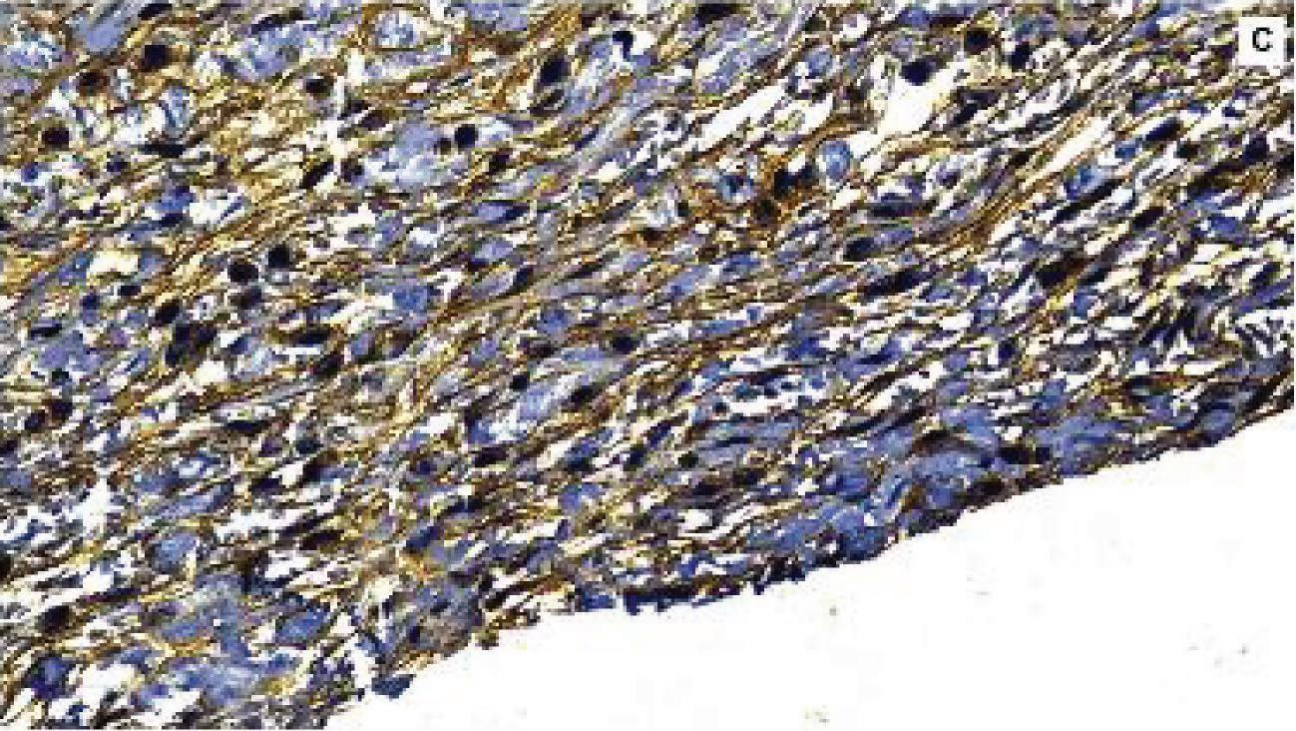
(c)

**Figure:**
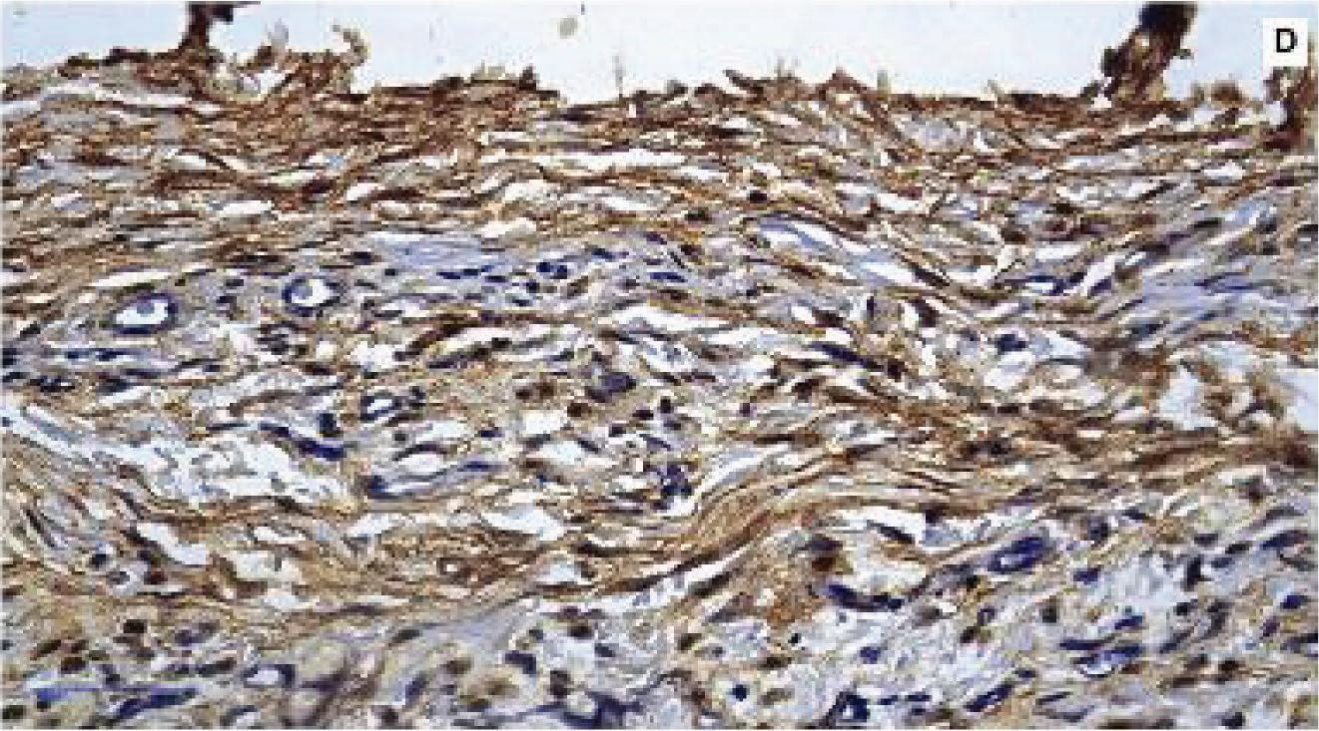
(d)

**Figure:**
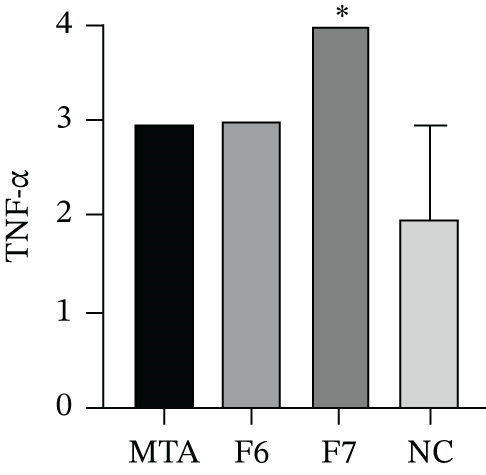
(e)

#### 3.2.5. Elemental and Molecular Characterization of Subcutaneous Tissues by Raman Spectroscopy and XRF

Raman and XRF analyses were employed to characterize the chemical composition of the tissue surrounding the implanted materials in samples collected at 30 days of experimental implantation (*n* = 3 samples per group and per time period). These techniques provided complementary molecular and elemental information regarding the distribution of chemical species associated with the experimental formulations F6 and F7, enabling the identification of calcium‐based compounds and radiopacifying elements at the tissue–material interface, as previously described for bioactive endodontic cements.

**Figure 7:**
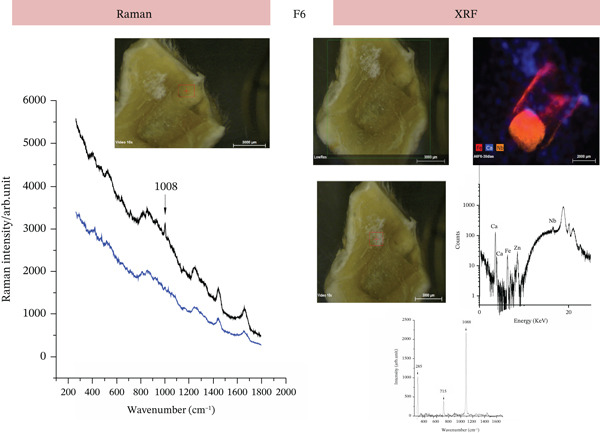
Raman and XRF analyses of tissue adjacent to the F6 implant (30 days). Raman panel (left): Raman spectra obtained from tissue adjacent to the F6 implant. The black and blue curves represent different sampling points. Peaks near 1086 cm^−1^ indicate the presence of calcium carbonate (CaCO3), whereas additional signals are compatible with the presence of collagen in the tissue. The inset shows the tissue sample during Raman acquisition. XRF panel (right): Macroscopic and micro‐XRF 2D maps of subcutaneous tissue after implantation of the F6 cement. The elemental map highlights the distribution of calcium (Ca) and niobium (Nb), with calcium deposits observed outside the implant area. The spectral data confirmed the presence of Ca and Nb in the tissue.

In the F6 group, XRF spectroscopy clearly revealed the presence of Nb within the tissue adjacent to the implant. Additionally, XRF analysis revealed a strong signal for calcium (Ca) distributed throughout the peri‐implant tissue, supporting the presence of mineralized regions (Figure 7b). Indeed, distinct Raman features corresponding to calcium sulfate (CaSO4) and calcium carbonate (CaCO3) were observed outside the implantation site, indicating mineral deposition or tissue calcification (Figure 7a). Importantly, the detection of Nb in the adjacent tissue is attributed to close proximity to the implant surface and interfacial interaction rather than to gross material extrusion, as no evidence of cement displacement or fragmentation was observed macroscopically or histologically. Together, these findings support the bioactive behavior of the F6 formulation and its ability to promote calcium compound deposition at the tissue interface [22].

**Figure 8:**
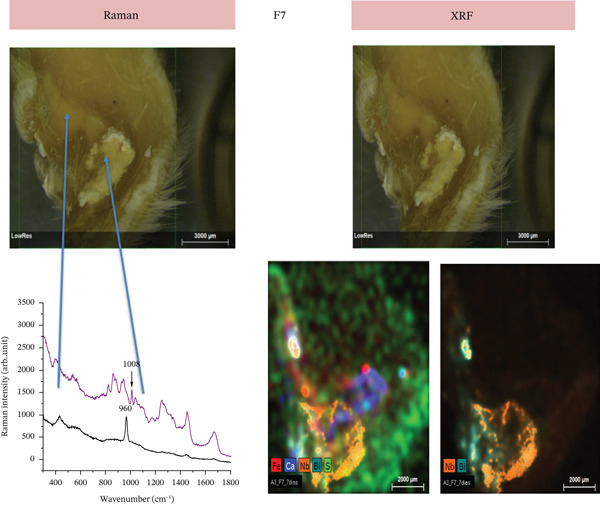
Raman and XRF analyses of tissue adjacent to the F7 implant (30 days). Raman panel (left): Raman spectra of tissue adjacent to the F7 implant, highlighting peaks associated with calcium sulfate (CaSO4) and apatite (Ca_10_(PO_4_)_6_) superimposed with the Raman features of collagen from the tissue. Two regions of interest (arrows) in the tissue are connected to the corresponding spectra (black and purple lines). XRF panel (right): XRF mapping and macroscopic image of the subcutaneous tissue after F7 implantation. Bismuth (Bi) and niobium (Nb) signals are concentrated mainly in the central implant area, whereas calcium (Ca) signals are more widely distributed in the surrounding tissue. Elemental overlays reveal strong localization of Bi, suggesting its retention in the implantation site as well as in a region demarked with a red circle.

In the F7 group, XRF mapping revealed the presence of bismuth (Bi) and niobium (Nb) localized within the implant area, along with a calcium‐rich outer region, suggesting chemical stratification or material diffusion into the surrounding tissue (Figure 8b). Raman spectroscopy confirmed the presence of calcium sulfate (CaSO4) in the tissue adjacent to the implant, together with apatite (Ca_10_(PO_4_)_6_) in the tissue covering the implant, which was consistent with the XRF findings (Figure 8a).

## 4. Discussion

This study evaluated the biological response to two experimental endodontic cements (F6 and F7) based on Portland cement incorporating different radiopacifying agents (Nb2O_5_ and bismuth oxide, respectively) using in vitro and in vivo analyses. Overall, the findings demonstrate that the F6 formulation containing niobium exhibited favorable biocompatibility and bioactivity, whereas the F7 formulation containing bismuth oxide had cytotoxic effects and a more pronounced inflammatory response.

Importantly, the integration of in vitro cell‐based assays with in vivo subcutaneous implantation allowed a comprehensive evaluation of the biological performance of the tested materials, enabling correlation between early cellular responses and subsequent tissue reactions.

The significantly reduced cell viability observed for the F7 formulation after 24 h compared with that of MTA suggests that a cytotoxic effect is likely associated with the inclusion of bismuth oxide. Although ALP activity remained similar across groups after 14 days, indicating preserved osteogenic potential, the early decrease in viability highlights the potential for acute cytotoxicity. These findings agree with those of Akhtar et al. [23], who demonstrated that bismuth oxide (Bi2O3) nanoparticles induced selective toxicity in human endothelial cells, primarily through mechanisms involving oxidative stress and disruption of the integrity of the cellular membrane. The parallel between their model and the response observed in MC3T3‐E1 preosteoblastic cells in our study supports the hypothesis that Bi^3+^ ions or particles released from the F7 formulation may exert similar toxic effects on osteogenic precursors.

The F6 formulation, which incorporates Nb2O_5_, demonstrated comparable cell viability and ALP activity to those of the MTA group. At 24 and 48 h, no statistically significant differences in MC3T3‐E1 cell viability were observed between F6 and MTA, suggesting a noncytotoxic profile. Similarly, after 14 days of osteogenic induction, the ALP activity of F6 remained similar to that of MTA, reinforcing its osteogenic potential. These findings are supported by Obata et al. [24], who reported that niobium ions released from calcium phosphate invert glasses containing Nb2O_5_ promoted osteoblast‐like cell functions, including proliferation and mineralization. Their study highlighted that niobium‐containing materials not only exhibit bioinert characteristics but also actively support bone‐related cell behavior, depending on their integration into the host matrix. In agreement, the F6 formulation in the present study appears to balance biocompatibility and osteoinductive capacity, positioning niobium oxide as a promising additive in endodontic cements.

Histopathological evaluation of the F7 formulation revealed an initial intense inflammatory response at 7 days, including the presence of multinucleated giant cells and high TNF‐*α* immunoexpression. These findings suggest pronounced immune activation in response to the material. This pattern is consistent with the study by Marciano et al. [25], who demonstrated that bismuth‐containing endodontic materials, such as MTA, can release Bi^3+^ ions into surrounding tissues in vivo, triggering the recruitment of macrophages and the formation of foreign body giant cells even 30 days after implantation. The authors also observed persistent bismuth deposits within the subcutaneous tissue, which correlated with chronic inflammation and delayed healing. In agreement with these data, the early upregulation of TNF‐*α* observed in our study indicates a sustained proinflammatory microenvironment potentially induced by the presence of bismuth oxide in F7, reinforcing the hypothesis that bismuth compounds may not be entirely inert in vivo and may contribute to adverse tissue responses.

F6 formulation, composed of Portland cement associated with Nb2O_5_ and hydrated calcium sulfate, demonstrated excellent in vivo biocompatibility in the present study. Histologically, F6 induced only mild to moderate inflammation at the early time period, with a significant reduction by Day 14 and minimal immune activity on Day 30. Importantly, no multinucleated giant cells were observed at any time period, and TNF‐*α* immunoexpression at 7 days was moderate and comparable to the response elicited by the MTA control, indicating that F6 did not trigger an exaggerated proinflammatory response. These findings are in line with those of the study by Mestieri et al. [26], who investigated the biological response of calcium silicate–based cements incorporating Nb2O_5_. In that study, the authors demonstrated that both the microparticulate and nanoparticulate forms of Nb2O_5_ exhibited low cytotoxicity in fibroblast‐ and osteoblast‐like cell lines (Saos‐2), showing comparable or even superior cell viability compared with conventional MTA. Additionally, materials containing Nb2O_5_ induced calcium deposition and supported the maintenance of cell function without triggering adverse reactions.

In addition to the favorable inflammatory and immunohistochemical profiles observed in the present study for the F6 formulation, our findings also highlight its mineralization potential and tissue integration capacity. Raman spectroscopy and XRF analyses revealed calcium deposits adjacent to the implantation site, along with the presence of niobium species in the surrounding tissue, which was consistent with a bioactive response. These observations align with those of the study by Silva et al. [12], who evaluated a calcium silicate–based cement containing niobium pentoxide (Nb2O_5_) and reported that the incorporation of this compound resulted in adequate radiopacity, alkaline pH, and enhanced mineral deposition while maintaining excellent biocompatibility in vivo, with no signs of toxicity or adverse reactions. In line with these findings, our histological analysis revealed the absence of multinucleated giant cells and only mild inflammatory infiltrates that progressively decreased over time. Moreover, PSR staining demonstrated increased collagen organization, reinforcing the notion of favorable tissue remodeling in response to F6. The convergence of histological, molecular, and elemental analyses strengthens the interpretation that F6 actively promotes tissue remodeling rather than acting as a passive biomaterial.

Together, these results support the hypothesis that Nb2O_5_ acts as a safe and effective radiopacifying agent that not only maintains the desirable physical and chemical properties of the cement but also contributes positively to the biological behavior of the material. Therefore, Nb2O_5_ stands out as a promising alternative to traditional radiopacifiers such as bismuth oxide, which has been associated with cytotoxicity and chronic inflammatory responses in vivo.

The osteogenic and mineralization‐promoting effects observed for the F6 formulation may be directly attributed to the presence of niobium pentoxide (Nb2O_5_) in its composition. Nb2O_5_ appears to act through both physicochemical and biological mechanisms. From a physicochemical standpoint, niobium oxide increases alkalinity and enhances calcium ion (Ca^2+^) release during the hydration process, creating a local microenvironment favorable for apatite nucleation and maturation [12, 13]. This is consistent with the Raman and XRF findings in the present study, which demonstrated calcium carbonate and calcium sulfate deposits adjacent to the implantation site, as well as niobium incorporation into the peri‐implant tissue. Biologically, niobium‐containing materials have been shown to stimulate osteoblast differentiation and upregulate genes associated with bone matrix formation, including ALP, osteocalcin, and Type I collagen. These effects are believed to result from niobium’s ability to modulate surface charge and enhance protein adsorption, favoring the attachment and maturation of osteogenic cells [24].

In agreement with this evidence, the F6 formulation supported early osteogenic activity in vitro, as demonstrated by preserved ALP activity after 14 days, and promoted in vivo collagen organization and calcium deposition, indicating a coordinated osteogenic and mineralization response. Therefore, Nb2O_5_ not only acts as a radiopacifying and structurally stable additive but also plays an active biological role in enhancing mineralized tissue formation and improving the regenerative performance of the cement. Thus, the preservation of ALP activity observed in vitro and the enhanced collagen organization observed in vivo appear to be biologically interconnected outcomes.

The favorable profile of F6 suggests that niobium pentoxide may serve as a viable alternative to traditional radiopacifiers, combining radiopacity with biocompatibility and bioactivity. This is further supported by previous findings from our group [14], in which the same formulation was evaluated and shown to exhibit adequate radiopacity according to ISO 6876 standards, as well as stable pH, dimensional stability, and calcium ion release. In that study, X‐ray microanalysis and Von Kossa staining confirmed the presence of calcium crystals in the subcutaneous tissue surrounding the implants, confirming the material’s bioactive potential [14]. These findings are consistent with previous in vivo evidence demonstrating that calcium silicate–based cements are capable of inducing biomineralization after subcutaneous implantation, as shown by Von Kossa–positive areas and calcium‐ and phosphorus‐rich surface precipitates detected by SEM/EDX analyses [27].

These physicochemical properties are directly aligned with the biological response observed in the present study, where F6 promoted minimal inflammatory infiltrates, the absence of multinucleated giant cells, and moderate TNF‐*α* expression, along with progressive collagen fiber organization over time. Together, these findings reinforce the concept that the incorporation of Nb2O_5_ not only ensures the radiographic detectability of the material but also supports a regenerative tissue response without triggering cytotoxic or proinflammatory effects. These results support the use of F6 as a next‐generation endodontic material capable of fulfilling both structural and biological demands in clinical applications.

In subcutaneous implantation models using polyethylene tubes, the formation of a collagen capsule is an expected foreign body response and occurs predominantly around the tube wall rather than at the open ends or surrounding any extruded material. In the present study, collagen deposition was consistently confined to the fibrous capsule surrounding the tube itself, with no evidence of collagen accumulation in the connective tissue adjacent to extruded material at any experimental time period, including at 30 days.

This finding is particularly relevant, as the absence of collagen deposition or fibrotic reaction around extruded materials indicates that the tested formulations did not induce additional connective tissue encapsulation beyond the response elicited by the carrier tube. Therefore, the progressive increase in collagen thickness and organization observed over time reflects normal capsule maturation and tissue remodeling rather than a material‐driven fibrogenic response. Collectively, these observations support the biological inertness and favorable biocompatibility profile of the evaluated materials.

This study used a subcutaneous model, which does not fully replicate the environment of the periapical tissues or root canals. Nevertheless, this model is widely accepted for preliminary biocompatibility screening and provides robust information regarding inflammatory behavior and tissue compatibility. Future studies should consider the use of models that simulate endodontic conditions more closely, such as periapical lesion models or direct pulp capping scenarios. In addition, long‐term evaluations and mechanistic studies (e.g., studies of oxidative stress markers and macrophage polarization) may clarify the immunological pathways involved.

The results of this study demonstrate that the experimental formulation F6, composed of Portland cement combined with Nb2O_5_, has a broadly favorable biological profile, with low cytotoxicity, induction of osteogenic activity, and a controlled inflammatory response both in vitro and in vivo.

## Author Contributions

R.V.S.A. contributed to data collection, experimental procedures, formal analysis, and writing (original draft preparation). R.A.S.S.F. contributed to data collection, experimental procedures, formal analysis, and writing (original draft preparation). V.G.S. performed experimental procedures, contributed to validation, and assisted with data analysis. C.A.G.B. contributed to methodology development, statistical analysis, and writing (review and editing). M.L.D.S.L. participated in data acquisition, laboratory analyses, and preparation of figures and tables. N.C.S. assisted in experimental assays, contributed to data curation, and participated in manuscript revision. V.S.A. conducted laboratory analyses, contributed to validation, and assisted in preparing the final manuscript. A.S.S. contributed to biochemical analyses, interpretation of results, and writing (review and editing). R.F.A.J. contributed to conceptualization, methodological design, supervision of experimental phases, formal analysis, validation, and critical revision of the manuscript. N.K.C. participated in project administration, formal analysis, and writing (review and editing). I.L.G.S. contributed to experimental procedures, formal analysis, validation, and critical review of the manuscript. P.C.M. contributed to experimental procedures, formal analysis, validation, and critical review of the manuscript. S.P. participated in experimental procedures and formal analysis and assisted in data interpretation and editing of the revised version. A.A.A. is responsible for conceptualization, supervision, funding acquisition, project administration, data interpretation, and writing (final review and approval of the manuscript).

## Funding

This work was supported by the Conselho Nacional de Desenvolvimento Científico e Tecnológico (https://doi.org/10.13039/501100003593; 303915/2023‐4 and 401672/2023‐9) and by iCEIS (406264/2022‐8).

## Ethics Statement

This study was approved by the Animal Ethics Committee of the Federal University of Rio Grande do Norte (UFRN), Brazil, under protocol number 267.037/2021.

## Conflicts of Interest

The authors declare no conflicts of interest.

## Data Availability

The experimental data reported in this work are available upon reasonable request by email to the corresponding author.
